# Performance of EuroSCORE II in Octogenarians Undergoing Coronary Artery Surgery (from the KROK Registry)

**DOI:** 10.3390/jcm13226863

**Published:** 2024-11-14

**Authors:** Grzegorz Hirnle, Sleiman Sebastian Aboul-Hassan, Piotr Knapik, Zdzisław Tobota, Bohdan Maruszewski, Jan Rogowski, Wojciech Pawliszak, Paweł Bugajski, Marek Deja, Tomasz Hrapkowicz, on behalf of the KROK Investigators

**Affiliations:** 1 Department of Cardiac, Vascular and Endovascular Surgery and Transplantology, Silesian Centre for Heart Diseases, Medical University of Silesia, 41-800 Zabrze, Poland; t.hrapkowicz@sccs.pl; 2 Department of Cardiac Surgery, Zbigniew Religa Heart Center “Medinet”, 67-100 Nowa Sol, Poland; s.aboul-hassan@inm.uz.zgora.pl; 3 Department of Cardiac Surgery and Interventional Cardiology, Faculty of Medicine and Medical Sciences, University of Zielona Gora, 65-417 Zielona Gora, Poland; 4 Department of Anesthesiology, Intensive Therapy and Emergency Medicine, Silesian Centre for Heart Diseases, Medical University of Silesia, 41-808 Zabrze, Poland; pknapik@sum.edu.pl; 5 Department of Paediatric Cardiothoracic Surgery, Children’s Memorial Health Institute, 04-730 Warszawa, Poland; ztobota@ecdb.pl.pl (Z.T.); bmar@ecdb.pl.pl (B.M.); 6 Department of Cardiac and Vascular Surgery, Medical University of Gdansk, 80-210 Gdańsk, Poland; janrog@gumed.edu.pl; 7 Department of Cardiac Surgery, Dr Antoni Jurasz Memorial University Hospital, 85-094 Bydgoszcz, Poland; w.pawliszak@cm.umk.pl; 8 Department of Cardiac Surgery, J. Struś Hospital, 61-285 Poznań, Poland; pawelbugajski@onet.eu; 9 Department of Cardiac Surgery, Medical University of Silesia, 40-055 Katowice, Poland; mdeja@sum.edu.pl

**Keywords:** coronary artery surgery, EuroSCORE II, hospital mortality, octogenarians, prediction

## Abstract

**Background:** Octogenarians constitute the fastest-growing segment within contemporary cardiac surgery, yet precise risk assessment in this age group remains challenging. **Aims:** This study aimed to evaluate EuroSCORE II reliability in octogenarians undergoing isolated coronary surgery and to create an adjustment formula if necessary. **Patients and Methods:** All octogenarians who had isolated coronary surgery in Poland from January 2012 to December 2023, recorded in the Polish National Registry of Cardiac Surgical Procedures (KROK registry), were retrospectively assessed. EuroSCORE II’s reliability was measured using the ROC curve area and observed-to-predicted mortality ratio, differentiating on-pump and off-pump cases. A nomogram was developed to enhance predictive accuracy. **Results:** Among 5771 octogenarians, 2729 (47.3%) underwent on-pump and 3042 (52.7%) underwent off-pump surgery. EuroSCORE II demonstrated reliability in off-pump patients (AUC:0.72, O/E ratio:0.98) but underestimated mortality for on-pump cases (AUC:0.73, O/E ratio:1.62). The lowest predicted mortality group (0.50–1.82%) showed the greatest discrepancies. Calibration was improved by adding a coefficient and creating a nomogram. **Conclusions:** EuroSCORE II was dependable in predicting outcomes for Polish octogenarians undergoing isolated coronary surgery. Observed mortality following on-pump surgeries was higher than expected, which was corrected by adding a coefficient to the initial EuroSCORE II calculation.

## 1. What’s New?

Cardiac surgery is currently confronted with a growing population of octogenarians with coronary artery disease and clear indications for coronary artery bypass grafting.

Mortality risk assessment has been widely used in cardiac surgery for many years. The European System for Cardiac Operative Risk Evaluation (EuroSCORE) did not meet all expectations, therefore an upgraded version—EuroSCORE II model was presented. Since then, EuroSCORE II has been widely used and tested globally. EuroSCORE II has exhibited reliability in octogenarians undergoing off-pump coronary artery bypass (OPCAB) procedures but has proved to be significantly unreliable in elderly patients undergoing on-pump procedures. Predicted mortality was found to be higher in this patient group and could be improved by our complex formula. Incorporating new variables into contemporary operative risk models, particularly frailty, porcelain aorta, chest wall malformation, or chest radiation should lead to more precise operative mortality assessment. We present a new nomogram that clinicians can use in their routine clinical practice.

## 2. Introduction

Octogenarians are the fastest growing part of the contemporary cardiac surgical population. With advancing age, these patients often present with increased comorbidities and complexities, making surgical outcomes more challenging to predict and manage. In the Polish population, off-pump coronary artery surgery (OPCAB) has been proved to be safer in comparison to conventional on-pump coronary artery bypass grafting (ONCAB) in this group of patients [1], reflecting a significant advancement in surgical techniques and patient safety.

Mortality risk assessment has been a cornerstone in cardiac surgery for many years, helping surgeons to better understand and mitigate the risks associated with complex procedures. The European System for Cardiac Operative Risk Evaluation (EuroSCORE) was first introduced in 1999 [2] and quickly became the most recognized predictive tool for cardiac surgeons in Europe. However, the initial EuroSCORE did not meet all expectations, prompting the need for modifications and adjustments. An upgraded model was introduced 12 years later, in response to these demands for a more accurate and reliable tool.

In 2011, the EuroSCORE II model was presented at the 25th Congress of the European Society of Cardiothoracic Surgery in Lisbon [3] and described in detail the following year [4]. Since then, EuroSCORE II has been widely used and tested globally, not only in Europe, providing a more refined assessment of surgical risk. Its broader application has underscored the importance of continuous evaluation and adaptation to diverse patient populations.

Two years following the launch of the EuroSCORE II model, a meta-analysis of 22 papers assessing the credibility and overall performance of this new score was published. The authors confirmed the good performance of this score, but it also became clear that validation in various populations will need to be the focus of future studies [5]. This highlights the dynamic nature of risk assessment tools and the necessity for ongoing research to ensure their relevance and accuracy across different demographic groups.

We hypothesized that adjusting the existing and popular mortality risk calculator would provide better prediction of hospital mortality in octogenarians undergoing coronary surgery. Therefore, the primary aim of our study was to assess the reliability of the EuroSCORE II surgical risk calculator in the group of octogenarians who underwent coronary artery surgery in Poland. The secondary aim of our study was to create an appropriate adjustment tool, if it proves necessary. This study seeks to enhance the predictive accuracy of EuroSCORE II, potentially leading to improved patient outcomes and more personalized surgical care for elderly patients.

## 3. Patients and Methods

This study is based on retrospective registry data from the Polish National Registry of Cardiac Surgical Operations: the KROK registry. This comprehensive registry has been gathering data from all cardiac surgical departments in Poland since 2006, providing a robust and extensive dataset for analysis. The rules of operation of the KROK registry have been described in detail elsewhere [6], ensuring transparency and reliability in data collection and processing.

All patients over 80 years old (n = 6228) who underwent coronary artery bypass surgery between January 2012 and December 2023 and whose data were collected in the KROK registry were analyzed. This large cohort allows for a thorough examination of surgical outcomes and risk factors specific to the octogenarian population. The extensive timeframe also ensures that trends and changes in surgical practices and patient outcomes can be accurately assessed.

All data processing was conducted under the protection of the electronic systems provided by the National Centre for Healthcare Information Systems. This ensures that the integrity and security of the data are maintained throughout the study. Additionally, all personal data were protected in accordance with Polish law, adhering to strict confidentiality and ethical standards. This rigorous approach to data management underscores the reliability and validity of the study’s findings, providing valuable insights into the outcomes of coronary artery bypass surgery in elderly patients.

### 3.1. Statement

The study utilized retrospective data from the Polish National Registry of Cardiac Surgery Procedures (the KROK registry), a comprehensive database covering all cardiac surgeries in Poland. Established through collaboration between the Polish Ministry of Health and the Polish Society of Cardiothoracic Surgeons, the registry is integrated with the National Health Fund, tracking nationwide mortality data since 2006. All data were anonymized, and the study complied with the Declaration of Helsinki. Ethics approval was granted by the Medical University of Silesia—Institutional Ethics Committee (PCN/0022/KB1/113/20), with all cardiac surgery departments submitting data to the National Centre for Healthcare Information Systems, under Ministry of Health oversight.

### 3.2. Study Population

The study is based on data from all 6228 octogenarian patients who underwent coronary surgery in Poland between 1 January 2012, and 31 December 2023. All patients analyzed were included in the KROK registry. The KROK registry allows one to assess the most important preoperative, intraoperative and postoperative variables for each patient. EuroSCORE II is calculated in the KROK registry for each patient since its introduction in January 2012.

Patients who underwent minimally invasive direct coronary artery bypass (MIDCAB) or a hybrid approach (457 patients, 7.3% of the whole population of octogenarians) were excluded from the study. The remaining 5771 patients were enrolled and divided into two groups. The first group consisted of 2729 patients operated on with the on-pump (ONCAB) technique (n = 47.3%) and the second group consisted of 3042 patients operated on with the off-pump (OPCAB) technique (n = 52.7%).

### 3.3. Statistical Analysis

For the primary aim, EuroSCORE II discrimination and accuracy were meticulously assessed. Receiver operating characteristics curve analysis (ROC) was used to estimate the performance of EuroSCORE II in predicting hospital mortality, a critical measure of the model’s effectiveness. Calibration of the model was assessed by the Hosmer–Lemeshow goodness-of-fit test, which evaluates how well predicted outcomes agree with actual outcomes. Additionally, the area under the curve (AUC) with a 95% confidence interval (CI) was calculated to quantify the overall ability of EuroSCORE II to distinguish between patients who survived and those who did not. The expected-to-observed operative mortality ratio (O/E) was calculated for the whole population and separately for each consecutive quartile of EuroSCORE II, providing a detailed insight into the model’s performance across different risk levels. All calculations were performed for the overall CABG cohort and separately in patients who underwent either off-pump or on-pump CABG, ensuring a comprehensive evaluation.

For the secondary aim, we assessed the performance of the original EuroSCORE II score specifically for octogenarians who underwent ONCAB or OPCAB. This analysis is crucial as it addresses the unique risk profile of elderly patients. Parameters of the function correcting the original EuroSCORE II to the ideal observed-to-expected mortality ratio (1.0) were determined using the least squares method, a statistical approach that minimizes the differences between observed and predicted values. A formula adjusting the original score was created, and a corresponding nomogram was drawn, providing a visual tool for clinicians to predict patient outcomes more accurately. To assess the performance of the EuroSCORE II score, the observed range of EuroSCORE II results was divided into quartiles, creating four operative groups with progressive operative risk (0.88–1.91%, 1.92–3.04%, 3.05–5.13%, and 5.14–maximum%). This stratification helps to identify how well the score predicts outcomes across different risk levels. Our methods of calculations have been previously described in detail in one of our previous studies, where we adjusted mortality risk prediction of the original EuroSCORE II for patients undergoing surgical reoperation for bleeding in the postoperative period [2].

Continuous variables were presented as mean and standard deviations, while categorical variables were presented as percentages, providing a clear and concise representation of the data. To test differences between the groups, Chi2, Mann–Whitney U, and Student’s *t*-tests were used, as appropriate, ensuring robust statistical analysis. For each analysis, a two-tailed *p*-value ≤ 0.05 was considered significant, underscoring the statistical rigor of the study. Analyses and graphs were undertaken using TIBCO Software Inc. (2017), Munchen, Germany, Statistica (data analysis software system), version 13, a sophisticated tool for statistical analysis and visualization. This comprehensive approach ensures the reliability and validity of our findings, contributing valuable insights into the optimization of surgical risk assessment for octogenarian patients.

## 4. Results

A total of 5771 patients over 80 years old who underwent isolated coronary artery surgery in Poland between January 2012 and December 2023 were analyzed. In this group, 2729 patients (47.3%) were operated on with the on-pump (ONCAB) technique and 3042 patients (52.7%) were operated on using the off-pump (OPCAB) technique.

**Figure 1:**
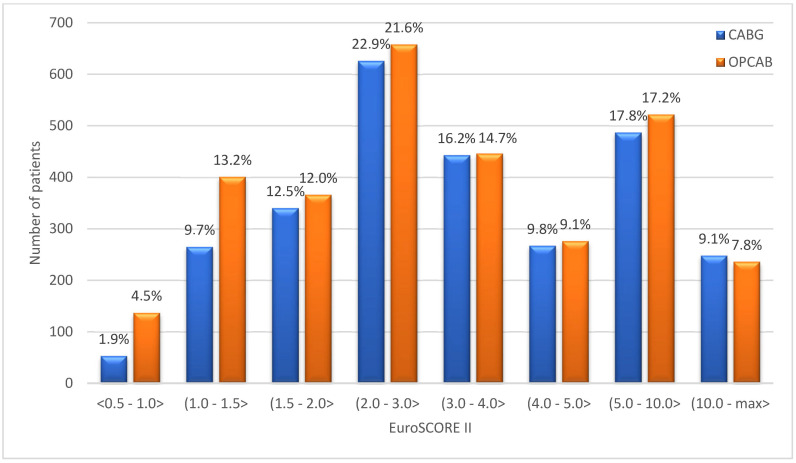
Distribution of EuroSCORE II in the analyzed population.

Figure 1 presents the histogram of predicted mortality based on EuroSCORE II. Most patients (71.1% operated on-pump and 70.6% operated off-pump) had a EuroSCORE II between 1% and 5%. The mean EuroSCORE II was higher in patients undergoing CABG (5.06 ± 6.52% vs. 4.61 ± 6.03%, *p* < 0.001).

**Figure 2:**
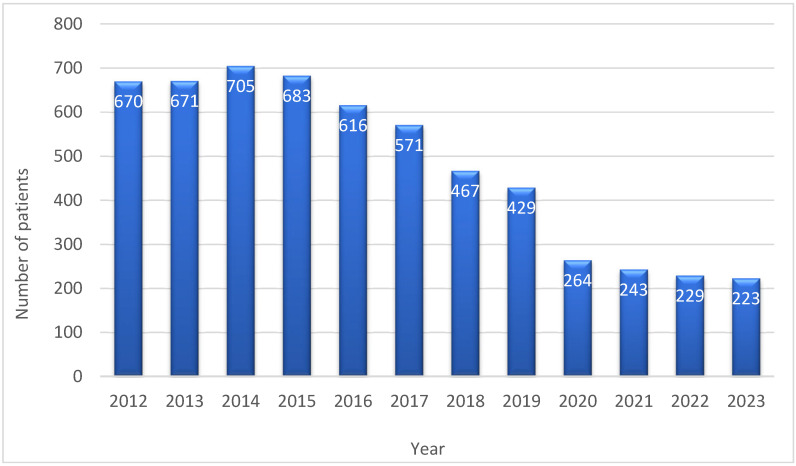
The number of coronary artery procedures (ONCAB and OPCAB) performed in octogenarian patients in the consecutive years during the observation period.

The number of procedures performed annually in the analyzed period was constant (Figure 2).

The mean age of all analyzed patients (both ONCAB and OPCAB) was 82.1 ± 2.1 years (from 80 to 96 years). Regarding demographic variables and preoperative comorbidities, significant differences were found between ONCAB and OPCAB patients. OPCAB patients were found to have significantly more comorbidities (Table 1).

The observed mortality of all octogenarians who underwent coronary artery surgery in Poland was 6.27%, while the predicted mortality was 4.82%, creating an observed-to-predicted mortality ratio (O/E) of 1.30. The distribution of the O/E ratio, however, was not equal (or even similar) among patients undergoing ONCAB and OPCAB. The observed mortality among octogenarians undergoing ONCAB was 8.21%, while the predicted mortality was 5.06% (O/E = 1.62). The corresponding figures for octogenarian patients undergoing OPCAB were 4.54 and 4.61 (O/E = 0.98), respectively.

In-hospital mortality among patients who underwent OPCAB was recorded at 4.54% (with 138 actual deaths), while EuroSCORE II anticipated a mortality rate of 4.61% ± 6.03%, estimating an average of 140 deaths (not statistically significant). The model demonstrated strong discriminatory power for identifying in-hospital mortality risk (AUC 0.72, 95% CI 0.68–0.77, *p* < 0.001) and was well-calibrated for this group (Hosmer–Lemeshow, *p* = 0.34), with an observed-to-expected (O/E) ratio of 0.98. Thus, EuroSCORE II proved effective for mortality prediction among OPCAB patients.

**Figure 3:**
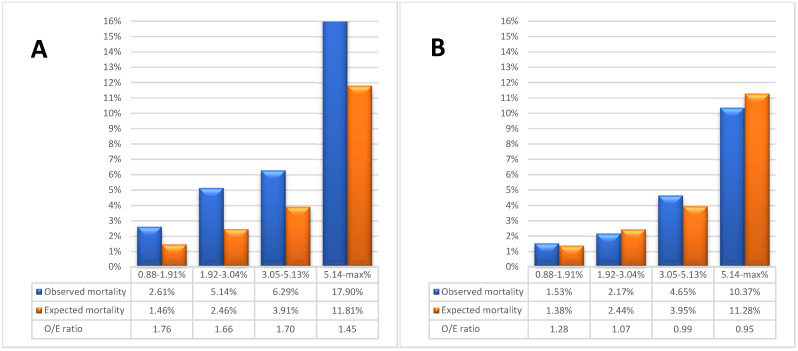
The results for each quartile of EuroSCORE II for patients undergoing ONCAB (**A**) and OPCAB (**B**).

In contrast, in-hospital mortality for ONCAB patients reached 8.21% (224 observed deaths), whereas EuroSCORE II predicted a lower mortality rate of 5.06% ± 6.52%, anticipating an average of 138 deaths (*p* < 0.001). Although the AUC value showed good discrimination in this subgroup (AUC 0.73, 95% CI 0.69–0.76, *p* < 0.001), the model lacked calibration for this population (Hosmer–Lemeshow, *p* < 0.001), with a higher O/E ratio of 1.62. In summary, while EuroSCORE II demonstrated reliable discrimination among octogenarians undergoing ONCAB, it significantly underestimated actual mortality in this subgroup, revealing a need for further adjustment to improve accuracy (Figure 3A).

The assessed performance of EuroSCORE II score in four operative groups with progressive operative risk (0.88–1.91%, 1.92–3.04%, 3.05–5.13%, 5.14–max%) is shown in Table 2.

### Prediction of Hospital Death with Adjusted EuroSCORE II

For patients undergoing ONCAB, EuroSCORE II demonstrated strong discrimination in predicting in-hospital mortality risk; however, its calibration was suboptimal. To address this discrepancy, a corrective function was developed by identifying parameters that refined the original EuroSCORE II score. Using the least squares method, adjustments minimized prediction errors, enabling a more accurate alignment of expected mortality rates with actual outcomes. This recalibration enhances EuroSCORE II’s precision, making it a more effective tool for assessing mortality risk specifically in the ONCAB patient population. The following formula for calculating the adjusted Euroscore II was created based on this formula:

mod ES II=ES II×(1.6545−0.0067×ES II)

The mortality rate observed in this cohort was 8.21% (224 deaths), while the adjusted EuroSCORE II predicted a mortality rate of 7.91% ± 9.01%, with an average of 216 expected deaths (*p* = 0.73). The AUC value of 0.73 (95% CI 0.69–0.76, *p* < 0.001) indicated strong discriminatory power for the SR population. The Hosmer–Lemeshow test confirmed good calibration for predicting in-hospital mortality (*p* = 0.87), yielding an O/E ratio of 1.01.

**Figure 4:**
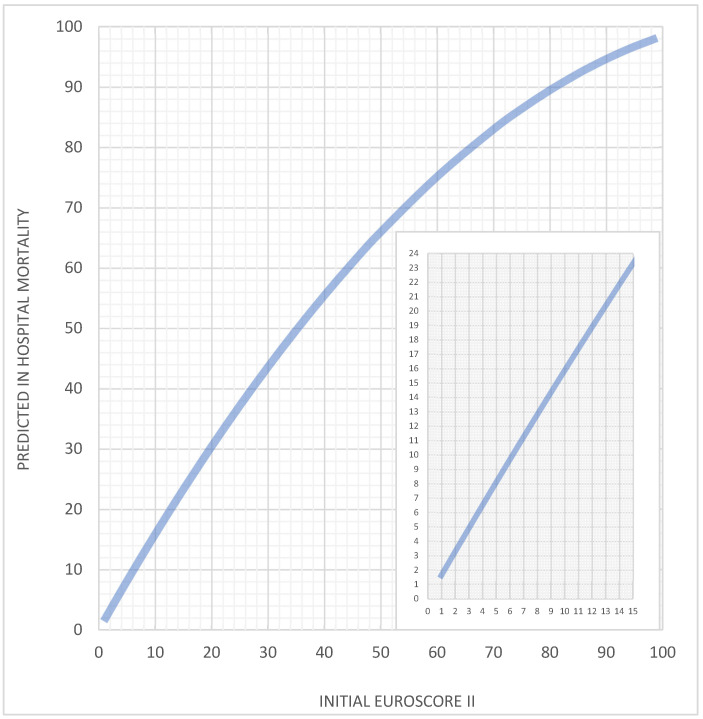
A nomogram of the calculations of predicted mortality.

Figure 4 presents a nomogram illustrating predicted mortality, where the original EuroSCORE II values on the horizontal axis correlate with calculated in-hospital mortality for ONCAB patients on the vertical axis. This tool allows for a straightforward, manual adjustment of initial mortality predictions to suit the risk profile of octogenarians undergoing ONCAB.

## 5. Discussion

This population-based study aimed to assess the discriminatory accuracy and calibration of the EuroSCORE II risk stratification tool in a nationwide cohort of octogenarians who underwent the CABG procedure. Despite the widespread use of EuroSCORE II for mortality risk assessment globally, it is evident that adjustments are required. With the anticipation that EuroSCORE II may become outdated, efforts have commenced towards the development of EuroSCORE III. Hence, the decision was made to initiate research by examining the general reliability of EuroSCORE II in octogenarians, utilizing real-word data from the KROK registry. It has been established already that risk assessment tools display reliable performance but underestimate mortality in Polish patients undergoing coronary artery surgery (KROK 2).

The estimated mortality rate was inaccurately low to varying degrees across different subgroups of the overall population. Knapik et al. and Boracci et al., divided the research cohort into quartiles and analyzed the observed-to-expected (O/E) ratio for each group [2,7]. This same methodology for mortality assessment was adopted in this study. The findings indicate that, at lower EuroSCORE II ranges (0.5–1.82%), the risk was underestimated, while accuracy improved at higher ranges (Figure 2). In the OPCAB, the risk was overestimated at higher EuroSCORE II ranges (4.82–68.33%), reaching an O/E ratio of 0.95. This divergence was not due to the proportion of missing data, as this percentage was consistent across all quartiles.

These outcomes were compared with the information available in the medical literature, yielding conflicting results. A meta-analysis encompassing 22 studies and 145,592 cardiac surgical procedures evaluated the reliability of EuroSCORE II [5]. For isolated coronary patients, EuroSCORE II generally overestimated mortality, with an O/E ratio of 0.804, yet the opposite occurred in the high-risk coronary surgery group [5].

Boracci et al. presented entirely different findings [7]. For isolated coronary artery procedures, the area under curve (AUC) was 0.794, and mortality was markedly underestimated (with an O/E ratio as high as 1.5). The authors attributed this to incomplete data in the medical registries, which are crucial for accurate EuroSCORE II estimation (e.g., left ventricular function, systolic pulmonary pressure, creatinine clearance) [7].

The discrepancies within medical literature should not deter us from critically analyzing available observational studies [8]. Hogervorst et al. [9] observed, that EuroSCORE II underestimated mortality in the Netherlands, Spain [10] and Canada [11], while it yielded satisfactory results in the UK [12]. EuroSCORE II proved to be unreliable for UK patients ≥70 years old [13], yet in Turkey and Canada EuroSCORE II displayed appropriate discrimination capacity in high-risk octogenarians [14,15]. Howell et al. noted that EuroSCORE II did not increase risk prediction in high-risk patients undergoing cardiac surgery compared with the original additive and logistic EuroSCOREs [16]. Certain significant factors, such as frailty, may not be adequately addressed in outcome prediction [17,18].

Speculations have arisen about replacing EuroSCORE II with a more accurate model. Several comparisons between EuroSCORE II and the American STS score have been conducted, revealing comparable performance [19].

As the risk of isolated coronary artery surgery steadily rises, predictive models require constant updates. A study by Therese K T Chua et al. underscored the increasing risk profiles of isolated coronary surgery [20]. Adapting models to perpetually evolving, diverse populations remain challenging. An imminent solution may come from a recent study where a machine learning model surpassed EuroSCORE II in predicting mortality after elective cardiac surgery [21]. Machine learning, involving computational statistics and predictions via computers, shows progressive enhancement in performance. Once machine learning and artificial intelligence (AI) are firmly integrated into medical practices, these challenges may fade. Each cardiac center could develop its continually updated predictive model. For now, EuroSCORE II remains the standard, and this study aimed to assess its reliability in octogenarians undergoing isolated coronary surgery.

A large study that involved 16,096 coronary surgery patients found an O/E ratio of 0.73. EuroSCORE II is a reasonable alternative to STS-PROM score in low-risk CABG patients (<5%) and in those undergoing other cardiac surgical procedures [22]. Otherwise, it overestimates operative risk. STS-PROM risk score was found to be more accurate in these circumstances, however it is not easy to compare those two risk calculators. Data for EuroSCORE II were gathered from 43 countries and data for STS-PROM score just from the US. Additionally, EuroSCORE II risk calculation needs 18 variables whereas STS-PROM requires over 40 [22].

A study based on 14,118 CABG patients’ data that involved 1349 octogenarians with a mean age of 82.2 (SD ± 2.0) showed interesting results. Patients were divided into three groups based on EuroSCORE II values: <4% (n = 895, 66.3%), 4–8% (n = 305, 22.6%) and >8% (n = 149, 11.0%). The results of this study remain in contradiction with our findings. The observed mortality in octogenarian patients undergoing coronary surgery was lower than expected throughout the whole EuroSCORE range [23].

In this study, hospital mortality in the whole population of Polish octogenarians undergoing coronary surgery reached 5.7%, whereas the predicted mortality was only 4.5%. While O/E was underestimated, the AUC indicated good discrimination in this group, with an AUC of 0.72. Mortality underestimation persisted across all quartiles, with the lowest reliability observed among patients with initially low expected mortality, ranging from 0.5% to 1.82% (where O/E ratio was as high as 1.48). EuroSCORE II demonstrated reliability and accurately predicted hospital mortality, especially in the second, third, and fourth EuroSCORE II quartile. In a subgroup of patients undergoing ONCAB, EuroSCORE II calibration was weakest in the lowest quartile.

A secondary objective of our study was to create a model for predicting perioperative mortality in octogenarians. To achieve this, we utilized advanced statistical methods and developed a complex formula to adjust the original EuroSCORE II. We present a nomogram that clinicians can use in their routine clinical practice.

A significant limitation of this study lies in its reliance on data solely from the medical registry, necessitating a retrospective design. Analyzing extensive data from such sources brings inherent challenges, like selection bias and missing data. It is important to mention that the data imported into the KROK database are heterogeneous. Furthermore, this study utilized registry data, which was strictly limited to the data available in KROK registry [2]. Additionally, the follow-up analysis was limited to all-cause mortality, which means that it is impossible to assess whether the patient’s death after discharge was related to the coronary artery bypass surgery or was due to an unrelated cause. Evidence-based medicine demands a receptive attitude and acknowledgment of its limitations [8]. The study’s strength lies in its extensive nationwide, real-world cohort, significantly contributing to our understanding of EuroSCORE II’s performance in octogenarians undergoing isolated CABG procedures and surgical risk.

In conclusion, EuroSCORE II exhibited reliability in octogenarians undergoing OPCAB procedures but proved significantly unreliable in elderly patients undergoing on-pump procedures. Predicted mortality was notably higher in the ONCAB patient group and could be improved by introducing a coefficient to the calculated EuroSCORE II. Incorporating new variables into contemporary operative risk models, particularly frailty, porcelain aorta, chest wall malformation, or chest radiation—factors of particular importance in elderly patients—should lead to more precise operative mortality assessment and enhance decision making in this critical patient population. In such specific patient groups, decision making should involve multidisciplinary heart-team discussions, amalgamating risk scores, clinical judgment, and additional variables.

## Figures and Tables

**Table 1:** Comparison of octogenarians undergoing ONCAB and OPCAB.

Variable	ONCAB (n = 2729)		OPCAB (n = 3042)		*p*
Age (years)	82.00	SD 2.08	82.20	SD 2.17	<0.001
BMI (kg/m^2^)	27.10	SD 3.83	27.17	SD 3.67	0.246
LVEF (%)	48.61	SD 13.02	48.75	SD 14.66	0.256
EuroSCORE II (%)	5.06	SD 6.52	4.61	SD 6.03	<0.001
Female gender	883	32.4%	1044	34.3%	0.121
CCS class IV	597	21.9%	634	20.8%	0.355
NYHA class III or IV	453	16.6%	569	18.7%	0.040
Recent MI < 90 days	1193	43.7%	1105	36.3%	<0.001
Pulmonary hypertension	12	0.4%	23	0.8%	0.169
LVEF < 30%	105	3.8%	129	4.2%	0.491
Previous PCA/stent	752	27.6%	688	22.6%	<0.001
Persistent or chronic AF	268	9.8%	353	11.6%	0.032
Left main stem lesion	1160	42.5%	1009	33.2%	<0.001
Triple vessel disease	1697	62.2%	1366	44.9%	<0.001
Cigarette smoking	96	3.5%	171	5.6%	<0.001
Hypercholesterolaemia	1710	62.7%	1787	58.7%	0.003
Diabetes mellitus	900	33.0%	1150	37.8%	<0.001
Arterial hypertension	2468	90.4%	2555	84.0%	<0.001
BMI > 35 kg/m^2^	73	2.7%	68	2.2%	0.320
Renal failure	836	30.6%	910	29.9%	0.572
COPD	264	9.7%	230	7.6%	0.005
Past TIA, RIND, stroke	113	4.1%	120	3.9%	0.756
Past treatment of CAD	36	1.3%	37	1.2%	0.817
PVD	444	16.3%	377	12.4%	<0.001
Poor mobility	242	8.9%	323	10.6%	0.029
Cardiogenic shock	118	4.3%	147	4.8%	0.391
Use of IABP	79	2.9%	35	1.2%	<0.001
i.v. nitrates or heparin	444	16.3%	420	13.8%	0.010
Previous cardiac surgery	36	1.3%	37	1.2%	0.817
Non-elective surgery	1367	50.1%	1544	50.8%	0.633

Abbreviations: AF—atrial fibrillation, CAD—carotid artery disease, CCS—Canadian Coronary Score, COPD—chronic obstructive pulmonary disease, IABP—intra-aortic balloon pump, IV—intravenous, LVEF—left ventricular ejection fraction, MI—myocardial infarction, NYHA—New York Heart Association, PCA—percutaneous coronary angioplasty, PVD—peripheral vascular disease, RIND—reversible ischaemic neurologic deficit, TIA—transient ischaemic attack.

**Table 2:** The observed-to-expected mortality ratio in octogenarians undergoing CABG, divided into quartiles of the observed EuroSCORE II range.

	ES II Quartile	n	Observed Mortality	Predicted Mortality	O/E Ratio
All	0.88–1.91%	1462	1.98%	1.41%	1.48
	1.92–3.04%	1440	3.61%	2.45%	1.35
	3.05–5.13%	1431	5.45%	3.93%	1.34
	5.14–83.14%	1438	14.12%	11.55%	1.20
ONCAB	0.88–1.91%	613	2.61%	1.46%	1.76
	1.92–3.04%	701	5.14%	2.46%	1.66
	3.05–5.13%	700	6.29%	3.91%	1.70
	5.14–83.14%	715	17.90%	11.81%	1.45
OPCAB	0.88–1.91%	849	1.53%	1.38%	1.28
	1.92–3.04%	739	2.17%	2.44%	1.07
	3.05–5.13%	731	4.65%	3.95%	0.99
	5.14–69.33%	723	10.37%	11.28%	0.95

## Data Availability

The raw data supporting the conclusions of this article will be made available by the authors on reasonable request.
